# Exploring the *Impact Factor*: Medical Students Mentoring High School Students and Cultivating Cultural Humility

**DOI:** 10.1089/heq.2017.0025

**Published:** 2018-03-01

**Authors:** Jordan Derck, Elizabeth Yates, Molly Kuo, Charles Hwang, William Sturdavant, Paula Ross, Jonathon Finks, Gurjit Sandhu

**Affiliations:** ^1^ University of Michigan Medical School, Ann Arbor, Michigan.; ^2^ Department of Surgery, University of Michigan, Ann Arbor, Michigan.; ^3^ Department of Learning Health Sciences, University of Michigan, Ann Arbor, Michigan.

**Keywords:** cultural humility, healthcare disparities, healthcare provider diversity, medical education, mentorship, underrepresented in medicine

## Abstract

**Purpose:** Diversity at all levels of medical training remains relatively stagnant, despite efforts to address equity in medical schools. Early career-specific mentoring may address barriers to the pursuit of medical education for students underrepresented in medicine (URiM). By surveying a program that engages medical students as drivers of career-specific mentorship for URiM high school students, this study evaluates medical student mentors' experiences mentoring and seeks to develop a mentorship curriculum.

**Methods:** The authors describe a medical student-led pipeline program, which connects medical students with URiM high school students. Medical student mentors participated in focus groups and gave written responses evaluating reasons for involvement, sociocultural attitudes, and skills needed for mentoring. Thematic analysis was applied.

**Results:** Themes that emerged in this analysis include motivation to mentor, skills used to approach the mentoring relationship, and benefits to the mentor. Mentors felt their experiences had a high impact factor, and they employed dynamic discovery. It provided personal reward and a deeper understanding of disparities.

**Conclusion:** Bringing medical school mentors together for peer to peer idea sharing, creating communities of practice, will help these students develop effective mentorship skills. A curriculum based on appreciative inquiry of mentors' strengths will enrich idea sharing, fostering cultural humility and avoiding burnout. Medical students involved in this program believe they gained benefits, including improving their mentorship skills, expanding their cultural humility, increasing their comfort with caring for underserved populations, and improving their ability to recognize health disparities.

## Introduction

There continues to be a lack of diversity at all levels of medical training and among practicing physicians, despite numerous initiatives to diversify the U.S. healthcare professional workforce.^1–4^ Efforts have focused on every level of medical training, including undergraduate pipeline programs, postgraduate baccalaureate programs, and medical school admissions.^5–7^ Each program seeks a common goal to expand the pool of underrepresented in medicine (URiM) students matriculating to medical school.

While the amount of success at changing the number of matriculates remains elusive, there have been significant advances in identifying barriers to pursuit of medical education by URiM students. Minority students have been provided with fewer academic resources than their peers before college.^8^ URiM students are subsequently less likely to pursue science majors in the first 2 years of undergraduate education.^9^ A smaller percentage will then pursue graduate education in science fields. In addition, of the students who choose premedical concentrations, a disproportionate number will experience lower grade point averages (GPAs) in premedical courses and lower Medical College Admissions Test (MCAT) scores.^10^ Some factors that have been associated with these findings are absence of career-specific mentors, challenges with navigating the academic environment, and the experiences and perceptions of interracial dynamics on campus.^3,6,10–13^

With these growing data, it is apparent that earlier interventions, specifically early career-specific mentorship, have the potential to address several of these barriers.^3^ The Doctors of Tomorrow program was founded in 2012 as a partnership between the University of Michigan Medical School (UMMS) and Cass Technical High School in Detroit, Michigan. At that time, more than 90% of students from Cass Tech self-identified as black or Hispanic, and only 9% of the UMMS student body self-identified with these underrepresented groups. Doctors of Tomorrow is centered on the potential benefits of medical students mentoring high school students. Previous analysis has demonstrated that the minority high school students involved in the Doctors of Tomorrow program feel that these mentoring relationships are transformative and critical in their pursuit for higher education in medicine.^14^

These mentoring relationships have the potential to foster cultural humility among both parties.^15^ In this context, cultural humility is defined as a respectful and inclusive attitude toward individuals who identify with cultures, races, and backgrounds different from one's own and pushes one to challenge his or her own biases, and to maintain mindfulness in one's relationships with others, particularly in the context of health disparities. To ensure that medical student mentors are able to foster appropriate mentoring relationships, it is important to understand the values and skills of the mentors. In addition, recognizing sociocultural values and strengths that medical student mentors bring to the Doctors of Tomorrow program helps shape a mentorship curriculum that capitalizes and expands upon those assets.

Taking an appreciative inquiry approach provides unique guidance in this evaluation. Appreciative inquiry is a process of discovery that seeks to apprehend and heighten positive potential.^16–18^ Evaluating this positive potential promotes positive change. Within the context of Doctors of Tomorrow, appreciative inquiry will be used to query the mentors' areas of strength in relationship to cultural attitudes and mentoring skills, which has the potential to inform the development of a curriculum to enhance and grow these preexisting traits. Fostering these skills in peer groups has the potential to build communities of practice among mentors.

Communities of practice are groups of people who share a concern or a passion for something they do and learn how to do it better as they interact regularly. When applied to mentorship, these communities cultivate more opportunities for growth by peer-to-peer idea sharing and learning, further cultivating cultural humility.^15,19,20^ Utilizing appreciative inquiry in communities of practice will enable mentors to reflect on and expand those qualities and strengths that are most advantageous in their role as mentors.

Using the potential barriers for URiM mentees described in the literature as a guide for our inquiry of medical student mentor social-cultural attitudes and mentoring skills, we implemented a project to create discussion among the mentors about their perception of their role in the mentoring relationship. The purpose of this report is to (1) identify what motivated medical students to get involved in the mentoring program, (2) explore awareness of health disparities and cultural humility among mentors, and (3) establish a foundation for developing a mentorship curriculum that is based on an appreciative inquiry model to capture the strengths and qualities of mentors and promotes mentor participation in communities of practice.

## Methods

### Rationale

The medical student mentors begin the Doctors of Tomorrow program each year with varying levels of experience mentoring and varying levels of exposure to diverse cultures. In the absence of a formal curriculum for mentors, it is likely that there is variability in the capacity for mentorship among medical students. It is crucial to the outcomes for the high school student mentees that their mentors feel appropriately equipped to guide the mentoring relationship. By creating a curriculum based on appreciative inquiry, the Doctors of Tomorrow program can maximize the positive mentorship potential of each medical student, enriching the mentoring relationships.

### Institutions

The mentoring program that is evaluated in this qualitative analysis was developed as a central component of the Doctors of Tomorrow program. Doctors of Tomorrow is a collaboration between the UMMS and Cass Technical High School in Detroit that was established to connect medical students with high school students who have an interest in medicine. The majority of Cass Technical High School students who participated in the program are black or African American, from low-income households, and with variable levels of parental education.^14^ The Doctors of Tomorrow program seeks to inspire these URiM students to pursue careers in healthcare and provide them with skills that will help them be more successful in that pursuit. The program also includes once-monthly visits by the students to the UMMS campus with a full day of career-specific activities, as well as a Capstone project and community outreach opportunities. The ultimate goal of this model is to improve diversity in the healthcare provider workforce by targeting students early in their education.

### Participants

The opportunity to be a medical student mentor has been presented to each first year UMMS class of ∼170 students. Ultimately, 17 students volunteered to be mentors in the initial year (2012–2013). The program has grown, with 35 first year medical students volunteering to be mentors for the 2015–2016 cohort. Each mentor is assigned a single mentee. The mentors who entered the program between 2012 and 2015 were invited to give feedback on their mentoring experiences through focus groups and written responses, with students from all 4 years of training providing feedback. From August 2015 to September 2015, 70 of the 135 medical student mentors either participated in focus groups or provided written feedback, giving a 52% response rate. Of those mentors, only 8 (11%) identified as black or Hispanic, and 40 (56%) identified as female. Involved mentors had demonstrated good participation in the program by at least attending required mentor time once per month, while mentees were visiting the medical school campus.

### The mentoring program

Upon volunteering for the mentorship program, first year medical student mentors attended four hours of orientation led by the Doctors of Tomorrow faculty advisor. The orientation covered program goals and mentor responsibilities; there was no specific focus on cultural humility at this stage. Mentors were consented for background checks (required by Cass Technical High School to work with their students). Mentors were expected to attend a one hour lunch and an hour-long Capstone project building session at UMMS with their mentees each month. In addition, mentors were invited to participate in all parts of the high school students' day at UMMS each month. There were three to five events held at Cass Technical High School, including Capstone presentations that mentors were strongly encouraged to attend. Mentors were additionally encouraged to contact their mentee at least once per month through email. This structure is enforced in the mentoring program through the first year of program involvement and mentors are encouraged to maintain communication as the mentor and mentee progress through medical school and high school, respectively. Mentoring is foundational to the program as it provides the ninth graders a near-peer guide and potential longitudinal support. As a part of their mentoring relationship, mentors are meant to provide information about the college and medical school experience along with insights on academic success strategies.

### Focus groups

Two focus groups were facilitated by one of the researchers (G.S.), with five medical students participating in one group and four in the other. The semistructured group interview process was developed and refined by three members of the research team. The group interview guide consisted of nine prompts that addressed reasons for involvement in the mentoring program, sociocultural attitudes, and skills needed for mentoring (see Supplementary Material). Each focus group lasted about 40 min. Each focus group was audio-taped and transcribed verbatim.

### Written responses

There was difficulty scheduling focus groups for medical students in their third and fourth years of medical school because of clinical duties. The feedback from these students was anticipated to be valuable in the data collection, given that they had been involved in the program the longest. It was decided that these students could give written feedback to the prompts used for the focus groups. A document with the nine focus group prompts was sent to all third and fourth year medical students, and they were invited to respond to any or all prompts. Sixty-one students provided written responses through email.

### Thematic analysis

All data were deidentified and J.D. individually coded the data using QSR NVivo11 (QSR International Pty Ltd., Doncaster, Australia). G.S. independently examined a subsection of the feedback and reviewed all of the thematic analysis. Discrepancies were reconciled among the study team members.

### Institutional review board approval

Ethical approval for this study was provided by the University of Michigan Institutional Review Board (IRB No. HUM000104321).

## Results

The medical student focus groups and written responses exposed three facets central to mentoring as medical students: why they mentor, what skills they use to approach the mentoring relationship, and how mentors benefit from the experience. In discussing their experiences mentoring, the medical students created a rich dialogue about the objectives that motivate them and the tools they need to be effective mentors.

### Theme 1: Action oriented motivation to mentor

Medical students often spoke about their motivations to get involved in service activities during medical school. They explained why they chose to mentor high school students, specifically URiM interested in healthcare. Students identified disparities and personal identity as considerations they reflected on when evaluating if the mentoring program would make a significant impact that aligned with their personal values. Other students also described personal previous experiences with disparities as motivation to participate. More broadly, they recognized the challenge that people of diverse backgrounds may experience in pursuing a medical degree (Table 1).

**Table 1.** **Medical Students' Comments on Their Motivations to Mentor**

Participant (sub-theme)	*Illustrative Quote*
Medical student 11	*Many of these opportunities provide only “one and done” interactions … Doctors of Tomorrow offered me an opportunity to develop a meaningful relationship with a high school freshman from Detroit who I could watch grow and mature over four years. The continuity of that kind of relationship is extraordinarily unique.*
Impact factor	
Medical student 4	*They [previous mentors] spoke about how well organized the program was and that the faculty leadership respected student voice. I was interested in a program that I could make an impact on rather than just being a moving part.*
Impact factor	
Medical student 18	*I was surprised when I got to the first day of medical school and looked around the class and it was not nearly as diverse as I thought that it would be.*
Addressing disparities	
Medical student 11	*There were countless people who helped me along the way in getting to medical school. Without their guidance and encouragement I never would have been able to arrive at this point in my life. Doctors of Tomorrow provided me with an opportunity to pay it forward and help young students in need of that kind of guidance and encouragement.*
Personal identity	
Medical student 08	*I've seen firsthand in my own family how not having educated parents with experience in higher education can make a student feel like school is out of reach for them (unfamiliarity with financial aid,* etc.).
Personal identity	

Citations from medical student focus groups and/or written responses that were associated with the theme of motivations to mentor, along with the medical student reference number and code that was affiliated with each comment, are included.

#### The impact factor

Medical students are necessarily selective in their participation in extracurricular and service activities because the demands on their time between academics and clinical duties are significant. Doctors of Tomorrow mentors highlighted the potential to make a tangible impact on their local community in the arenas of diversity and disparities as the primary motivator for dedicating their time to this mentorship program. They specifically highlighted long-term direct contact with mentees, student leadership, and proximity of the community targeted as important components that contribute to the “impact factor” of the program (Table 1).

#### Addressing disparities

Many medical students identified disparities in their own medical school class as motivation to participate in a program that addresses disparities in medicine. Students identified the lack of diversity in their class as a motivating factor (Table 1).

#### Personal identity

Medical students discussed personal identity when evaluating reasons to become mentors. They identified their own personal struggles while pursuing a medical degree. Many medical students adopted a “paying it forward” mentality when they reflected on their identity by trying to emulate the figures that helped them along the way (Table 1).

### Theme 2: Intuitively building a mentoring relationship

Medical students described their approach to building mentoring relationships with mentees. They recalled applying dynamic discovery or learning by doing, in addition to adopting traits from their role models (Table 2).

**Table 2.** **Medical Students' Comments on Building the Mentoring Relationship**

Participant (sub-theme)	*Illustrative Quote*
Medical student 22	*My mentee was pretty shy at the beginning, and so it was trying to find different approaches, trying to get to know her better, figure her out and see what kind of brought out her passion and what made her want to talk.*
Dynamic discovery	
Medical student 23	*The thing that helped me the most being a DOT mentor, but I think that definitely still had a really big influence on me [was] in terms of who my formal and informal mentors have been throughout my life and the ones that I felt made a really big impact on me and the ones that maybe didn't and why that might have been.*
Role-modeling	

Citations from medical student focus groups and/or written responses that were associated with the theme of building the mentoring relationship, along with the medical student reference number and code that was affiliated with each comment, are included.

#### Dynamic discovery

Mentors, especially those without previous mentoring experiences, often employed a “learning by doing” approach when trying to build effective mentoring relationships. They identified strategies they could use to engage with the high school students and evaluated which were effective. Mentors conveyed that this was a not systematic process that they had intentionally applied, but rather the intuitive response when approaching the mentoring relationship. Interestingly, this approach was shared by the majority. Mentors found mentoring to be challenging and experienced a sense of fulfillment when their strategies proved successful (Table 2).

#### Role-modeling

When mentors approached their relationship with mentees, they identified their previous experiences in the mentee role and used those experiences as models. They identified qualities in their previous mentors that were beneficial for making a connection. The mentors they identified were from formal mentoring programs and informal relationships (Table 2).

### Theme 3: Personal growth and changing trajectory

Mentors found that their work in the mentoring program was personally rewarding and often provided validation for the work they were putting into other areas of medical school. Mentors realized personal growth and broke down biases by their long-term interactions with mentees (Table 3).

**Table 3.** **Medical Students' Comments on Personal Growth**

Participant (sub-theme)	*Illustrative Quote*
Medical student 19	*It gave me more confidence in myself because I saw the trust that they placed in me.*
Personal reward	
Medical student 2	*… medicine can be a tough, draining lifestyle so you have to take responsibility for your own experience and be proactive in finding activities that help you avoid getting jaded. For me, DoT was the perfect organization that fit that bill.*
Personal reward	
Medical student 04	*I do feel like Doctors of Tomorrow was really good for my personal growth and exposure to different kinds of students and the barriers and experiences they have.*
Understanding disparities	
Medical student 20	*It gives me additional motivation to seek out people whose backgrounds are more challenging … it makes me want to reach out more.*
Understanding disparities	

Citations from medical student focus groups and/or written responses that were associated with the theme of personal growth, along with the medical student reference number and code that was affiliated with each comment, are included.

#### Personal reward

Mentors often described that the preclinical years of medical school can be challenging because they do not interact with patients. The demanding preclinical curricular schedule combined with the lack of tangible results has the potential to demoralize medical students. Mentoring, and community service in general, provides validation, which combats potential burnout. Mentors appreciate that the high school students are aspiring to similar goals that they themselves have already achieved. Mentoring can provide an initial experience for aspiring physicians, in which they feel they have gained more trust from an individual because of the hard work they have applied to studying medicine (Table 3).

#### Understanding disparities

Mentors felt that they gained a better understanding of disparities and the various barriers that individuals from URiM backgrounds experience after participating in the Doctors of Tomorrow program. Respondents identified disparities as creating barriers both to students pursuing medical education and to patients seeking healthcare (Table 3).

## Discussion

Our analysis of the medical student reflections about mentorship identifies the impact factor as a significant motivator for participation. We have used the term impact factor to describe the gauge medical students used when considering how to choose extracurricular activities. Using the impact factor, medical students considered how much impact they could make toward a cause of their interest. The students who chose mentorship through Doctors of Tomorrow were interested in specific issues, such as the lack of diversity in their medical school class, healthcare disparities in local communities, and a lack of career-specific mentoring for young underrepresented students seeking matriculation to medical school.

The mentors also discussed aspects of the program that led them to believe it had a strong impact factor. The mentors identified opportunities for student leadership and fluidity of the program as allowing for a strong mentor voice. In addition, the vicinity of the partnering community program, both by location and objective, creates a strong sense of proximal impact for the mentors. By identifying the tenets of the mentorship program that are important to the mentors, those components can be expanded upon to better serve the objectives of current and future mentors.

In a similar manner, the mentors identified skills and tools that they used to maximize their relationships with mentees. The mentors discussed tools that helped them improve their mentorship skills; they most often used dynamic discovery and role-modeling. Having identified these tools will help build an appreciative inquiry-based curriculum that will augment the mentors' experience and their mentoring potential. Bringing mentors together to participate in a curriculum will encourage peer-to-peer idea sharing and building of communities of practice. Likewise, inviting mentors who felt they have learned from strong role-modeling or developed effective strategies through dynamic discovery to share their experiences will benefit all mentors. Appreciative inquiry reinforces exploring and expanding upon pre-existing positive qualities and tools present in the mentors.

In designing a mentorship curriculum, the outcomes associated with participation in the mentorship program that mentors discussed could be potentiated. Mentors spoke about feeling validation and personal reward, as well as using the program as a tool to avoid burnout. As these conversations develop, there is the potential that working through some of these intellectual and sensitive issues in communities of practice will give mentors yet another outlet to help avoid the potential burnout. These conversations may include insights from their experiences with mentors and mentees, and may include mentors' own personal identity with respect to underrepresented backgrounds. In addition, they spoke about gaining a better understanding of disparities and breaking down biases. Communities of practice engender the opportunity to share experiences and understandings focused on disparities and diversity, thereby deepening cultural humility. As a process for self-reflection and self-evaluation, these practices become habitual in one's service and work with underserved communities. With more exposure to the impact that their work is having, mentors may be more likely to participate in efforts to reduce disparities in their future medical careers.

There are several limitations to this study. First, the Doctors of Tomorrow program is a single partnership between one medical school and one high school. This could restrict the variation in types of mentoring relationships that were fostered and limit the generalizability of our results. Second, participants may have expressed a more favorable opinion about mentoring because of their commitment to the Doctors of Tomorrow program. We had a robust sample size and explained to respondents that focus group transcripts and written responses would be deidentified in and attempt to mitigate this influence. Due to difficult medical student schedules, two different data sources had to be collected to attain a large sample size, which could also cause some variation in response. In addition, no data regarding the sociocultural attitudes of mentors were collected before participation in the Doctors of Tomorrow program. The results of the study represent the benefits of the program as perceived by the mentors. Pre-comparisons and post-comparisons of cultural humility would have helped augment our results. Finally, the focus of this study was on mentors. Inclusion of matching data collected from the high student mentees will inform future research on mentorship.

When considering the next steps for this research, our group has initiated the development of curriculum sessions for the mentors, which take into account the findings from this project. Mentors are required to participate in 50% of the sessions offered. The sessions have been presented in the form of interactive lunch talks dispersed throughout the academic year, which are facilitated by a physician or educator. Each session highlights a specific topic. Topic selection has been informed by the mentors' responses analyzed in this report. Sessions have included unconscious bias; communication; teaching on the fly; compassion fatigue; and coaching and mentoring. Highlights from the curriculum include the unconscious bias session, which integrates awareness about personal identity, cultural humility, and leveraging the benefits of diversity. The compassion fatigue session brings to the fore the need to care for oneself to optimally invest in the success of others (e.g., patients and mentees). The coaching and mentoring session provide actionable strategies for engaging with mentees and establishing responsibilities for both individuals in the relationship. These sessions and the rest of the curriculum have been well received by the mentors. Our future research will include further developing this curriculum, guided by our growing understanding of skills many mentors demonstrate during their participation in the Doctors of Tomorrow program.

## Conclusion

Ultimately, using the values identified by mentors in the study, including the impact factor, may help guide the development of this mentorship program and others. This analysis will enable the creation of a curriculum that provides maximal benefit to both medical student mentors and high school mentees by supporting mentor development. Ultimately, this curriculum provides significant benefits not only in form of an improved mentorship experience for the mentee but also to the medical student mentor in avoiding burnout, strengthening cultural humility, and building skills to address disparities in the future.

## Supplementary Material

Supplemental data

## Abbreviations Used

UMMS: University of Michigan Medical School
URiM: underrepresented in medicine

## References

[1] American Association of Medical Colleges (AAMC). Diversity in Medical Education: Facts & Figures 2012. Washington, DC, 2012

[2] AAMC. Diversity in the Physician Workforce: Facts and Figures 2014. Washington, DC, 2014

[3] AAMC. Altering the Course: Black Males in Medicine. Boston, MA, 2015

[4] Person SD, Jordan G, Allison JJ, et al. Measuring diversity and inclusion in academic medicine: the diversity engagement survey. Acad Med. 2015;90:1675–1683 26466376 10.1097/ACM.0000000000000921 PMC5823241

[5] Lakhan SA Diversification of U.S medical schools via affirmative action implementation. BMC Med Educ. 2003;3:6 13678423 10.1186/1472-6920-3-6 PMC212493

[6] Veazy BD, Ovink SM More Than “Getting Us Through”: a case study in cultural capital enrichment of underrepresented minority undergraduates. Res High Educ. 2011;52:370–394 24954971 10.1007/s11162-010-9198-8 PMC4059344

[7] Figueroa O The significance of recruiting underrepresented minorities in medicine: an examination of the need for effective approaches used in admission by higher education institutions. Med Educ Online. 2014;1–9 10.3402/meo.v19.24891 PMC4156603 25192970

[8] Perna LW, Swail WS Pre-college outreach and early intervention. Thought Action. 2001;17:99–110

[9] National Science Foundation, National Center for Science and Engineering Statistics. Women, minorities, and persons with disabilities in science and engineering. 2013 Available at www.nsf.gov/statistics/wmpd Accessed 715, 2016

[10] Alexander E, Grumbach K How leaky is the health career pipeline? Minority student achievement in college gateway courses. Acad Med. 2009;84:797–802 19474563 10.1097/ACM.0b013e3181a3d948

[11] Hurtado S, Han JC, Saenz VB, et al. Predicting transition and adjustment to college: biomedical and behavioral science aspirants' and minority students' first year of college. Res High Educ. 2007;48:841–887

[12] Villarejo M, Barlow AE, Kogan D, et al. Encouraging minority undergraduates to choose science careers: career paths survey results. CBE Life Sci Educ. 2008;7:394–409 19047426 10.1187/cbe.08-04-0018 PMC2592049

[13] Freeman BK, Landry A, Trevino R, et al. Understanding the leaky pipeline: perceived barriers to pursuing a career in medicine or dentistry among underrepresented-in-medicine undergraduate students. Acad Med. 2016;91:987–993 26650673 10.1097/ACM.0000000000001020

[14] Derck J, Zahn K, Finks JF, et al. Doctors of tomorrow: an innovative curriculum connecting underrepresented minority high school students to medical school. Educ Health. 2016;29:259–265 10.4103/1357-6283.204219 28406112

[15] Tervalon M, Murray-Garcia J Cultural humility versus cultural competence: a critical distinction defining physician training outcomes in multicultural education. J Health Care Poor Underserved. 1998;9:117–125 10073197 10.1353/hpu.2010.0233

[16] Carter CE, Ruhe MC, Weyer S, et al. An appreciative inquiry approach to practice improvement and transformative change in health care settings. Qual Manag Health Care. 2007;16:194–204 17627214 10.1097/01.QMH.0000281055.15177.79

[17] Quaintance JL, Arnold L, Thompson GS What students learn about professionalism from faculty stories: an “Appreciative Inquiry” approach. Acad Med. 2010;85:118–123 20042837 10.1097/ACM.0b013e3181c42acd

[18] Sugarman HC Appreciative Inquiry More Than a Methodology: Practitioners' Framing for Successful Transformation. Washington, DC: The Center for Association Leadership, 2002

[19] Wenger E Communities of practice and social learning systems: the career of a concept. In: Social Learning Systems and Communities of Practice. Edited by Blackmore C London: Springer, 2010, pp. 179–198

[20] Wenger-Trayner E, Wenger-Trayner B Introduction to communities of practice. 2015 Available at http://wenger-trayner.com/introduction-to-communities-of-practice Accessed 315, 2016

