# Supplementary material for: Exploring the Impact Factor: Medical Students Mentoring High School Students and Cultivating Cultural Humility
Source: Health Equity. 2018 Mar 1;2(1):15–21. doi: 10.1089/heq.2017.0025 (PMC6071896; doi:10.1089/heq.2017.0025)
Supplement: Supplemental data [file Supp_Data.pdf]

Supplementary Material

Discussion Guide for Focus Groups and Written Responses

1. Out of the numerous opportunities available to medical students, why did you choose the Doctors of Tomorrow Program at University of Michigan Medical School?
2. Why did you initially want to be in the Doctors of Tomorrow Program?
3. What prior experiences did you have working and/or volunteering with underserved or disadvantaged populations?
4. What does it mean to you when I say, “medically underserved communities”?
5. Specifically, what experiences have you had with medically underserved communities?
6. What experiences have you had working with people from diverse backgrounds, cultures, and ethnicities?
7. What experiences have you had with teaching, coaching, and/or mentoring?
8. What skills from your past will help you as a mentor in the Doctors of Tomorrow program?
9. What are some of your professional goals after completing residency?
